# Impact of Room Location on UV-C Irradiance and UV-C Dosage and Antimicrobial Effect Delivered by a Mobile UV-C Light Device

**DOI:** 10.1017/ice.2016.35

**Published:** 2016-03-23

**Authors:** John M. Boyce, Patricia A. Farrel, Dana Towle, Renee Fekieta, Michael Aniskiewicz

**Affiliations:** 1 Quality Improvement Support Services, Yale–New Haven Hospital, New Haven, Connecticut; 2 Yale University School of Medicine, Yale–New Haven Hospital, New Haven, Connecticut; 3 Department of Laboratory Medicine, Yale–New Haven Hospital, New Haven, Connecticut

## Abstract

**OBJECTIVE:**

To evaluate ultraviolet C (UV-C) irradiance, UV-C dosage, and antimicrobial effect achieved by a mobile continuous UV-C device.

**DESIGN:**

Prospective observational study.

**METHODS:**

We used 6 UV light sensors to determine UV-C irradiance (W/cm^2^) and UV-C dosage (µWsec/cm^2^) at various distances from and orientations relative to the UV-C device during 5-minute and 15-minute cycles in an ICU room and a surgical ward room. In both rooms, stainless-steel disks inoculated with methicillin-resistant *Staphylococcus aureus* (MRSA), vancomycin-resistant *Enterococcus* (VRE), and *Clostridium difficile* spores were placed next to sensors, and UV-C dosages and log_10_ reductions of target organisms achieved during 5-minute and 15-minute cycles were determined. Mean irradiance and dosage readings were compared using ANOVA.

**RESULTS:**

Mean UV-C irradiance was nearly 1.0E-03 W/cm^2^ in direct sight at a distance of 1.3 m (4 ft) from the device but was 1.12E-05 W/cm^2^ on a horizontal surface in a shaded area 3.3 m (10 ft) from the device (*P*<.001). Mean UV-C dosages received by UV-C sensors located at different distances and orientation relative to the device varied significantly during 5-minute cycles and during 15-minute cycles (*P*<.001). Log_10_ reductions ranged from >4 to 1–3 for MRSA, >4 to 1–2 for VRE and >4 to 0 log_10_ for *C. difficile* spores, depending on the distance from, and orientation relative to, the device with 5-minute and 15-minute cycles.

**CONCLUSION:**

UV-C irradiance, dosage, and antimicrobial effect received from a mobile UV-C device varied substantially based on location in a room relative to the UV-C device.

*Infect Control Hosp Epidemiol* 2016;37:667–672

Suboptimal cleaning of patient rooms at the time of discharge has generated substantial interest in the use of automated “no-touch” systems to reduce the risks of transmission of healthcare-associated pathogens.^1^ ^–^ ^3^ Mobile continuous ultraviolet light (UV) devices that emit primarily UV-C in the range of 254 nm have been shown to reduce bacteria on surfaces by 1–5 log_10_ depending on the device, cycle time, and type of pathogen.^4^ ^–^ ^14^ Several studies have found that the level of reduction of bacteria on surfaces may be greater when surfaces are in direct sight of such devices than when surfaces receive indirect light.^4^ ^–^ ^6^ ^,^ ^10^ Although this finding is assumed to be due to differences in the dosage of UV-C received by various surfaces, there is a paucity of published data on the impact of UV-C dosages delivered to surfaces in various locations and orientations in hospital rooms on the eradication of pathogens. We conducted a prospective study to measure UV-C irradiance and dosage levels and their effects on artificially contaminated disks exposed to an automated, mobile, continuous UV-C light device.

## METHODS

### UV-C Light Measurements

UV-C light measurements were obtained using model ILT254 radiometric UV-C light sensors (International Light Technologies, Peabody, MA) equipped with calibrated National Institute of Standards and Technology (NIST)-traceable UV-C detectors with 254-nm filters and wide-angle diffusers. UV-C irradiance (W/cm^2^) readings were recorded using Data Logger software provided by the manufacturer. UV-C dosage (Wsec/cm^2^) was determined at each sensor by adding all irradiance readings obtained every second during a cycle. UV-C dosage readings were subsequently converted to µWsec/cm^2^. UV-C irradiance and dosage were measured using several protocols. To assess the variability of UV-C readings obtained with the light sensors placed at different distances and in various orientations relative to the UV-C device, triplicate readings were obtained with sensors in fixed positions (ie, readings were taken during 3 cycles without moving sensors) and 25 minutes between cycles. We conducted 5-minute and 15-minute fixed-position cycles in an intensive care unit (ICU) room 29.16 m^2^ (324 sq ft) in size and in a room 27 m^2^ (300 sq ft) in size on a standard surgical ward. Rooms have standard wall paint, Marmoleum floors, dropped ceiling tiles, standard hospital beds, and in the ICU, physiologic monitoring devices. Temperature and humidity readings were not obtained. Rooms are maintained at temperatures between 68° and 75°F and at relative humidity of 30%–60%. The ICU room has 12 air changes per hour (ACH), and the surgical ward room has 6 ACH.

We placed 6 UV-C sensors simultaneously in different locations in a patient room. To promote standardized methods for evaluating the performance of mobile UV-C devices, we placed sensors at distances (1.2 m [4 ft] and 3.3 m [10 ft]) utilized by other investigators.^12^ ^,^ ^14^ We also evaluated UV-C irradiance and dosages achieved when sensors were oriented at 0° angles relative to the device to mimic dosage received by horizontal surfaces in hospital rooms. First, 2 sensors were oriented vertically and pointed directly at the UV-C device at distances of 1.3 m and 3.3 m from the device, then 2 sensors were oriented at a 0° angle to light emitted from the device (sensors lying flat on surfaces and pointed at ceiling) at distances of 1.3 m and 3.3 m. Finally, 2 sensors were oriented at a 0° angle to the light source and in shaded sites (indirect light) at distances of 1.3 m and 3.3 m. For each of the latter 2 experimental conditions, an opaque object oriented vertically was placed immediately next to the sensor and between the sensor and light coming directly from the UV-C device. No sensors were placed at a 90° angle opposite the UV-C device.

In addition, during a 15-minute cycle, qualitative photochromic UV-C indicator labels provided by the device manufacturer were placed on the radiometric UV-C sensors to assess color changes at varying dosages. The labels, which are initially white, develop increasingly darker shades of pink as the dosage received increases.

### UV-C Light Device

We utilized a newly built, automated, mobile, continuous UV-C device (Model 1000, Spectra 254, Danbury, CT) comprised of 8 64-inch high-output low-pressure UV-C bulbs that are designed to last 10 years The device is activated using a wireless remote control, which allows the user to choose a 5-minute, 10-minute, or 15-minute cycle.

To determine whether any antimicrobial effects might be attributed to generation of ozone by the UV-C device, ozone concentrations in each room were measured using a portable ozone monitor (Series 200 monitor, Aeroqual, Aukland, New Zealand) with a lower limit of detection of 5 ppb before and within 1 minute of ending decontamination cycles.

### Preparation of Inocula

Clinical strains of methicillin-resistant *Staphylococcus aureus* (MRSA) and vancomycin-resistant *Enterococcus* (VRE), and *Clostridium difficile* strain ATCC 9689 were used as target organisms. MRSA and VRE isolates were inoculated onto blood agar plates and incubated overnight at 37°C. For these 2 organisms, colonies from blood agar were used to make separate suspensions equivalent to a 0.5 McFarland standard in normal saline for inoculation onto disks. *C. difficile* spores were produced by inoculating the organism onto 10 horse-blood-agar plates (Remel, Lenexa, KS), which were incubated anaerobically for 5–7 days at 37°C. The plates were then held at room temperature for 7–10 days in a biological safety cabinet (BSC). After 7 days, spores were transferred to a tube containing 10 mL sterile dH2O and absolute alcohol (50/50 concentration) and stored at 4°C for further use.

### Microbiology

For both MRSA and VRE, 10 µL suspensions were inoculated over the entire surface of sterilized stainless-steel disks (1 cm diameter; Muzeen and Blythe, Winnepeg, Canada) and allowed to dry at room temperature in a BSC. For *C. difficile*, 300 µL spore suspension was centrifuged at 489 g for 5 minutes in a microcentrifuge. The supernatant was decanted and resuspended in 100 µL phosphate-buffered saline (PBS). The suspension was vortexed, and 10 µL of the suspension was then spread over the entire surface of stainless-steel disks. The inoculum was allowed to dry at room temperature under a hood. For each UV-C cycle, each target organism was inoculated onto 3 control disks that were not exposed to UV-C. Additional disks, each inoculated with a target organism, were placed on pieces of double-sided adhesive tape affixed to sterile Petri dishes. Petri dishes containing inoculated disks were attached to or placed immediately adjacent to sensors to determine the antimicrobial effect of the UV-C dosage received at each of the 6 sensor positions.

UV-C measurements were obtained on 3 days using 5-minute cycles and on 3 days using 15-minute cycles. On each day, 6 sensors were placed at the same distances and orientations relative to the device as those described above. For 1 of the 15-minute cycles, photochromic dosage indicator labels were attached to sensors to correlate color changes with dosages determined by radiometric UV-C light sensors.

Following exposure of test disks to UV-C treatment, each set of test and control disks was placed in 1,000 µL of PBS and vortexed. Serial 10-fold dilutions were made by adding 100 µL to 900 µL PBS followed by vortexing for 15 seconds, and 100 µL of each dilution was then inoculated onto appropriate agar media. Colony counts were determined after incubation for 48 hours for MRSA and VRE and 96 hours for *C. difficile*.

### Statistical Analysis

ANOVA methods were used to compare mean irradiance values and standard deviations obtained during 5-minute cycles in the ICU room and in the room on the surgical unit from triplicate fixed-position readings of the 6 sensors. To determine whether the large number of irradiance readings analyzed might have affected ANOVA results, Cohen’s *d* and effect size *r* values were calculated because these parameters are not affected by sample size. Mean dosages obtained during triplicate fixed-position 5-minute and 15-minute cycles were also compared using ANOVA methods. Log reductions of bacteria or spores achieved following exposure to UV-C were determined by subtracting colony counts obtained from disks exposed to UV-C from mean colony counts obtained from control disks not exposed to UV-C.

## RESULTS

### UV-C Light Irradiance and Dosages

**FIGURE 1:**
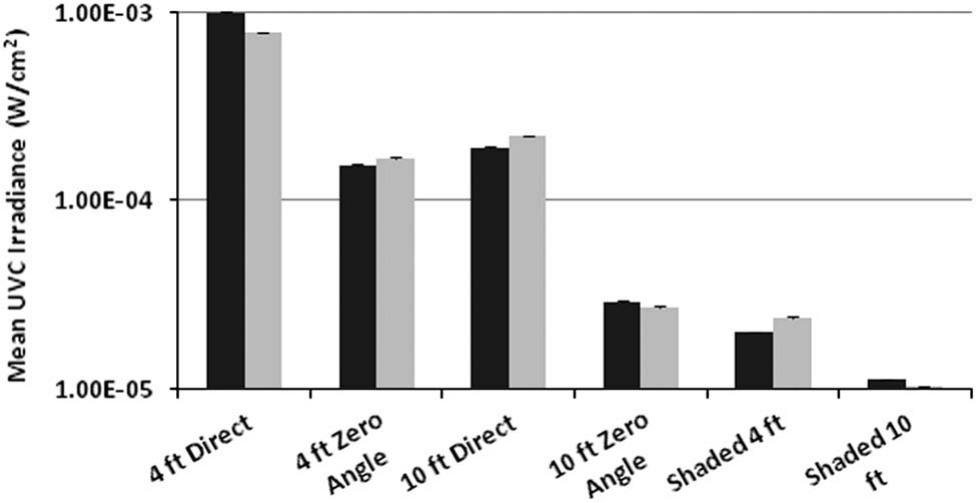
Mean ultraviolet C (UV-C) and standard deviation (SD) irradiance levels (W/cm^2^) measured with 6 UV-C sensors placed at different distances and orientations relative to a mobile UV-C device in an intensive care unit room (left column at each position) and a room on a surgical ward (right columns). Sensor positions included in direct sight at distances of 1.3 m (4 ft) and 3.3 m (10 ft), at a 0° angle relative to the device at distances of 1.3 m and 3.3 m, and in shaded areas 1.3 m and 3.3 m from the device.

The means and standard deviations of triplicate UV-C irradiance measurements obtained in fixed positions in the ICU room and the room on the surgical ward are shown in Figure 1. In the larger ICU room, mean irradiance was highest (9.89E-04 W/cm^2^ or nearly 1E-03 W/cm^2^) when a sensor was pointed directly at the device at a distance of 1.3 m and in direct sight of the device. Mean irradiance was lowest (1.12E-05 W/cm^2^) when a sensor was located on a horizontal surface 3.3 m from the device and in a shaded area. Irradiance levels achieved at the different room locations and positions were significantly different (*P*<.001). Similarly, with a 5-minute cycle performed in the somewhat smaller patient room, mean irradiance was highest (7.74E-04 W/cm^2^) when a sensor was pointed directly at the device at a distance of 1.3 m and in direct sight of the device; mean irradiance was lowest (1.03E-05 W/cm^2^) when a sensor was located on a horizontal surface 3.3 m from the device and in a shaded area. Irradiance levels at the different positions were significantly different (*P*<.001). As expected, mean UV-C irradiance levels measured in triplicate at 1.3 m and 3.3 m for 15-minute cycles in the 2 different rooms were very similar to those obtained during 5-minute cycles (data not shown). All pairwise comparisons of irradiance levels measured in the ICU room and in the patient room on the surgical floor yielded large effect-size *r* values (≥0.977 for all) when tested using Cohen’s *d*.

Mean UV-C dosages received by sensors located in 6 fixed positions during 5-minute cycles in each of the 2 patient rooms are shown in the Table 1. The highest mean UV-C dosage (358,667 µWsec/cm^2^) was achieved in the ICU room at a distance of 1.3 m with the sensor pointed directly at the device and in direct sight. At a distance of 3.3 m and with a sensor pointed at the ceiling and with indirect UV-C light, mean dosages were only ~4,000 µWsec/cm^2^ in the ICU room and <3,800 µWsec/cm^2^ in the room on the surgical ward. Dosages achieved at the 6 sensor positions varied significantly from one another in each of the 2 rooms (*P*<.001 for both rooms).

**TABLE 1:** Mean UV-C Dosages (µWsec/cm^2^) Received by Sensors Located at 6 Positions in an ICU Room and in a Room on a Standard Surgical Ward (Non-ICU Room)^a^

UV-C Sensor Distance from Device	Orientation of UV-C Sensor	5-Min Cycle, ICU Room, Mean Dosage, µWsec/cm^2^ (SD)	5-Min Cycle, Non-ICU Room, Mean Dosage, µWsec/cm^2^ (SD)	15-Min Cycle, ICU Room, Mean Dosage, µWsec/cm^2^ (SD)	15-Min Cycle, Non-ICU Room, Mean Dosage, µWsec/cm^2^ (SD)
1.3 m (4 ft)	90° angle, direct sight	358,667 (5,131)	280,667 (577)	750,667 (4,041)	819,667 (6,110)
1.3 m	0° angle, direct sight	55,867 (586)	60,533 (351)	152,333 (1,528)	169,333 (1,527)
1.3 m	0° angle, indirect light	7,223 (70)	8,713 (60)	21,400 (346)	27,000 (360)
3.3 m (10 ft)	90° angle, direct sight	69,233 (987)	80,300 (100)	205,000 (1,000)	247,667 (1527)
3.3 m	0° angle, direct light	10,533 (115)	9,733 (367)	28,767 (404)	35,267 (611)
3.3 m	0° angle, indirect light	4,047 (21)	3,737 (49)	8,407 (59)	11,767 (153)

UV-C, ultraviolet C; ICU, intensive care unit; SD, standard deviation.

a Triplicate fixed-position readings were obtained in each room with 5-minute and 15-minute cycles.

**FIGURE 2:**
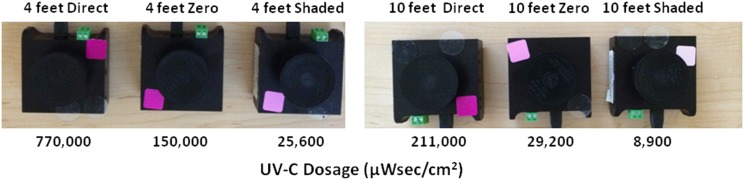
Results obtained with qualitative photochromic dosage indicator labels (red, pink, and white rectangles attached to sensors) and dosages (µWsec/cm^2^) measured using radiometric ultraviolet C (UV-C) light sensors placed at various distances from and orientations relative to the UV device after a 15-minute cycle. UV-C light normally enters the radiometric sensors through openings that were covered by circular elevated caps at the time photographs were taken.

Mean UV-C dosages received by sensors located in 6 fixed positions during 15-minute cycles varied significantly in each of the 2 patient rooms (*P*<.001) (Table 1). The maximal mean dosage recorded during 15-minute cycles (819,667 µWsec/cm^2^) was observed when a sensor in the patient room on the surgical ward was located 1.3 m from the device and was pointed directly at the device. At a distance of 3.3 m and in a shaded area, mean dosages were substantially lower, ranging from ~8,400 µWsec/cm^2^ in the ICU room and 11,767 µWsec/cm^2^ in the room on the surgical ward. Dosages achieved at the 6 sensor positions during 15-minute cycles varied significantly from one another in each of the 2 rooms (*P*<.001 for both rooms). Qualitative photochromic dosage indicator labels provided by the manufacturer turned dark pink with high dosages, light pink with intermediate dosages, and remained white with very low dosages delivered during a 15-minute cycle (Figure 2).

### Effects of 5-Minute and 15-Minute UV-C Cycles on Targeted Pathogens

The range of log_10_ reductions of MRSA, VRE and *C. difficile* achieved with 3 5-minute cycles and 3 15-minute cycles is shown in Table 2. With 5-minute cycles, 4 log_10_ reductions or more of MRSA were achieved at all locations and orientations except in a shaded area 3.3 m from the device, where 1–3 log_10_ reductions were achieved. The 5-minute cycles yielded reductions of VRE of 4 log_10_ or more when disks were located 1.3 m in direct sight of the UV-C device, with lower reductions achieved at 3.3 m (Table 2). The 5-minute cycles yielded reductions of *C. difficile* of 1 to 3 log_10_ when disks were facing the UV-C device or were at a 0° angle relative to the device at 1.3 m and at 3.3 m, but no detectable log_10_ reductions were achieved when disks were in a shaded areas 1.3 m or 3.3 m from the device (Table 2).

**TABLE 2:** Range of Log_10_ Reductions of MRSA, VRE, and *Clostridium difficile* Achieved with Inoculated Disk Carriers Exposed to UV-C for 5-Minute and 15-Minute Cycles on 3 Occasions at Each Cycle Time

Distance and Orientation of Disks Relative to UV-C Device	Mean UV-C Dosage Measured Adjacent to Disks for 5-Min Cycles, µWsec/cm^2^	Range of Log_10_ Reduction with 5-Min Cycles, by Pathogen	Mean UV-C Dosage Measured Adjacent to Disks for 15-Min Cycles, µWsec/cm^2^	Range of Log_10_ Reduction with 15-Min Cycles, by Pathogen
1.3 m (4 ft), direct	342,667	MRSA: >4 log	842,000	MRSA: >4 log
		VRE: 4 to >4 log		VRE: >4 log
		*C. difficile*: >2–3 log		*C. difficile*: 2 to >4 log
1.3 m, 0° angle	53,900	MRSA: 4 to >4 log	148,667	MRSA: >4 log
		VRE: 3 to >4 log		VRE: 3–4 log
		*C. difficile*: 1–2 log		*C. difficile*: 2–4 log
1.3 m, shaded	8,547	MRSA: 1–4 log	24,467	MRSA: >4 log
		VRE: 2–3 log		VRE: 2–3 log
		*C. difficile*: 0		*C. difficile*: 1–2 log
3.3 m (10 ft), direct	67,567	MRSA: 4 to >4 log	202.667	MRSA: >4 log
		VRE: 3 to >4 log		VRE: >4 log
		*C. difficile*: 1–3 log		*C. difficile*: 2–4 log
3.3 m, 0° angle	10,767	MRSA: 4 to >4 log	29,000	MRSA: 4 to >4 log
		VRE: 2 log		VRE: 3 log
		*C. difficile*: 0–1 log		*C. difficile*: 0–2 log
3.3 m, shaded	3,395	MRSA: 1–3 log	8,880	MRSA: 3 log
		VRE: 1–2 log		VRE: 1–2 log
		*C. difficile*: 0		*C. difficile*: 0–1 log

MRSA, methicillin-resistant *Staphylococcus aureus*; VRE, vancomycin-resistant *Enterococcus*; UV-C, ultraviolet C.

With 15-minute cycles, counts of MRSA on disks were reduced by 3 to >4 log_10_ and VRE by 1–4 log_10_ at varying distances and orientations relative to the UV-C device (Table 2). Log_10_ reductions of *C. difficile* were highest (2 to >4 log_10_) when disks were facing the device at a distance of 1.3 m and were lowest (0–1 log_10_) when disks were in a shaded area 3.3 m from the device (Table 2).

### Ozone Concentrations

Ozone levels measured in the larger ICU room were 0.015–0.017 ppm before and 0.014–0.018 ppm after a 5-minute cycle (no detectable ozone generation). In the smaller patient room on a surgical ward, ozone concentrations were 0.028–0.031 ppm before and 0.023–0.026 ppm at the end of a 5-minute cycle.

## DISCUSSION

The present study provides unique information regarding the UV-C irradiance levels and UV-C dosages that are achievable in patient rooms using a mobile continuous UV-C device. In contrast to a study by Rutala et al,^11^ which utilized a single UV-C sensor, we simultaneously placed 6 UV-C sensors in fixed positions in patient rooms and examined the degree of variability in UV-C irradiance levels obtained. We demonstrated that significant differences in irradiance levels are achieved when sensors are placed at various distances and orientations relative to light emitted from the device. For any given distance and orientation relative to the device, irradiance readings varied by 5%–6% in fixed positions, which was within the performance levels provided by the UV-C sensor manufacturer.

Despite considerable interest in UV-C mobile devices and adoption of this technology by some hospitals, we are not aware of data published in peer-reviewed journals regarding the UV-C dosages achieved with continuous UV-C devices in healthcare facilities. Our observations obtained in direct sight of the device at a distance of 1.3 m during a 5-minute cycle yielded UV-C dosages (ie, ~300,000 µWsec/cm^2^) and antimicrobial effects that were consistent with claims made by the UV-C device manufacturer. Log_10_ reductions observed in the present study are in the range reported by other investigators who also inoculated test pathogens onto stainless-steel disks.^12^ Not surprisingly, we found that shaded areas received significantly lower dosage levels than positions in direct sight of the device when single cycles were used. In other studies where continuous UV-C devices that measure the amount of light reflected back from different parts of the room to determine cycle time were tested, reduction of bacterial counts in shaded areas has often been somewhat, but not significantly, lower than those achieved in areas in direct sight of the device.^6^ ^,^ ^8^ ^,^ ^9^ ^,^ ^12^ ^,^ ^14^ In the present study, dosages measured immediately adjacent to inoculated disks (Table 2) demonstrate the impact of UV-C dosage on log_10_ reductions of pathogens on inoculated disks. Our findings provide new evidence to explain why log reductions of bacteria achieved in earlier studies were lower when inoculated surfaces were located at greater distances from UV-C devices and in shaded areas. Our inability to detect ozone generation may have been due to the relatively high rates of room air ventilation.

Our study has several limitations, including measurement of UV-C irradiance and dosages in only 2 rooms in a single hospital. Because we placed sensors in only 6 positions relative to the device, irradiance and dosage levels recorded may not reflect those achieved in other parts of the rooms. We did not study dosages received in various parts of test rooms that would have been achieved if rooms had been subjected to 2 or 3 consecutive cycles of UV-C treatment with the device placed at different locations in the room, as recommended by the manufacturer. Also, we did not evaluate the effect that different levels of organic material might have had on log reductions of pathogens achieved or whether our use of stainless-steel disks, which can reflect UV-C, may have yielded different log reductions than those achievable on other surfaces. No assessments for potential adverse effects on plastics were conducted. Furthermore, because irradiance and dosages were determined using a single, mobile, continuous UV-C device, our findings cannot be considered representative of all such devices. Other systems currently available differ in the type and size of UV-C bulbs utilized, type of reflective surfaces behind bulbs, and methods for monitoring UV-C dosage. Finally, we did not evaluate the ability of the UV-C device to reduce bacterial levels on high-touch surfaces or to reduce healthcare-associated infections.

In conclusion, measurements obtained using radiometric UV-C sensors revealed that irradiance and dosage levels measured at a distance of 1.3 m in direct sight of the device were consistent with claims made by the device manufacturer. However, UV-C irradiance, UV-C dosage, and antimicrobial effect achieved in patient rooms varied significantly, depending on the location and orientation of surfaces relative to the UV-C device. Such variations support recommendations by the device manufacturer to run >1 UV-C cycle in patient rooms. Similar variations in irradiance and dosage are likely to occur with use of other mobile continuous UV-C devices currently marketed. Additional studies to determine irradiance and dosages achieved by UV-C devices from other manufacturers may assist hospitals in device selection. Further studies are also indicated to establish the relationships between UV-C dosages and reduction of environmental contamination and healthcare-associated infections.
